# Cost of Acute Malnutrition Treatment Using a Simplified or Standard Protocol in Diffa, Niger

**DOI:** 10.3390/nu15173833

**Published:** 2023-09-01

**Authors:** Bernardette Cichon, Noemi Lopez Ejeda, Pilar Charle Cuellar, Issa Ango Hamissou, Ali Amadou Abdoul Karim, Cornelia Aton, Atté Sanoussi, Nassirou Ousmane, Ramatoulaye Hamidou Lazoumar, Abdoul Aziz Ousmane Gado, Zakou Yassi Harouna, Saul Guerrero Oteyza

**Affiliations:** 1 Action Against Hunger UK, London SE10 0ER, UK; 2 EPINUT Research Group (Ref. 920325), Unit of Physical Anthropology, Department of Biodiversity, Ecology and Evolution, Faculty of Biological Sciences, Complutense University of Madrid, 28040 Madrid, Spain; noemilop@ucm.es; 3 Action Against Hunger Spain, C/Duque de Sevilla no. 3, 28002 Madrid, Spain; 4 Independent Consultant, Niamey, Nigermirak3ak1993@gmail.com (A.A.A.K.); 5 Nutrition Direction, Ministry of Health, Niamey BP 623, Niger; at_sanoussi8@yahoo.fr (A.S.);; 6 Centre de Recherche Médicale et Sanitaire (CERMES), Niamey BP 10887, Niger; lramatoulaye@yahoo.fr; 7 Action Against Hunger, Niamey BP 11491, Niger; 8 UNICEF, 3 United Nations Plaza, New York, NY 10017, USA; sguerrerooteyza@unicef.org

**Keywords:** acute malnutrition, RUTF, cost, cost-efficiency, treatment

## Abstract

Evidence on the cost of acute malnutrition treatment, particularly with regards to simplified approaches, is limited. The objective of this study was to determine the cost of acute malnutrition treatment and how it is influenced by treatment protocol and programme size. We conducted a costing study in Kabléwa and N’Guigmi, Diffa region, where children with acute malnutrition aged 6–59 months were treated either with a standard or simplified protocol, respectively. Cost data were collected from accountancy records and through key informant interviews. Programme data were extracted from health centre records. In Kabléwa, where 355 children were treated, the cost per child treated was USD 187.3 (95% CI: USD 171.4; USD 203.2). In N’Guigmi, where 889 children were treated, the cost per child treated was USD 110.2 (95% CI: USD 100.0; USD 120.3). Treatment of moderate acute malnutrition was cheaper than treatment of severe acute malnutrition. In a modelled scenario sensitivity analysis with an equal number of children in both areas, the difference in costs between the two locations was reduced from USD 77 to USD 11. Our study highlighted the significant impact of programme size and coverage on treatment costs, that cost can differ significantly between neighbouring locations, and that it can be reduced by using a simplified protocol.

## 1. Introduction

Over the past two decades, the introduction of the community-based management of acute malnutrition model for treatment has enabled a shift in treatment of acute malnutrition from a purely facility-based approach to a decentralised community-based treatment provided by health centres. This approach has been implemented by many governments and non-governmental organisations to increase availability and access to treatment for children affected by acute malnutrition. However, over two thirds of the estimated 45 million children suffering from acute malnutrition [1], which increases the risk of death up to 12-fold in severe cases [2], do not currently have access to treatment [3].

In order to further increase coverage, there has been a growing interest in simplified approaches to treatment which aim to make treatment cheaper, simpler and even more easily accessible. These approaches, which include decentralising treatment to the village level, involving family members in the detection of malnutrition, combining treatment of moderate and severe acute malnutrition, altering dosage of ready-to-use therapeutic food (RUTF) or using a mid-upper arm circumference (MUAC)-only protocol for admission and discharge, have previously been shown to be both effective [4,5,6,7,8] and cost-effective [4,9,10].

When deciding on the best way to treat malnutrition in a particular setting and to improve cost-efficiency, evidence on the cost of treatment as well as the drivers of those costs is key. While evidence on cost and cost-effectiveness of approaches to treat acute malnutrition exists, it is very limited and difficult to compare due to different costing methods used [11]. Furthermore, treatment costs and cost-effectiveness are significantly influenced by context, local costs, malnutrition rates, population density, programme size, national treatment protocols and coverage [11,12]. Increasing the evidence base on the cost of malnutrition treatment across contexts is therefore important.

In 2021, Action Against Hunger, in collaboration with the Centre de Recherche Médicale et Sanitaire in Niger (CERMES), the Nutrition Division of the Ministry of Health in Niger and EPINUT research group from the Complutense University of Madrid (Spain), conducted an operational research project comparing effectiveness of acute malnutrition treatment with a standard and simplified protocol decentralised to the village level in an emergency context in Diffa region, Niger. In this study, the recovery rate among children treated with a simplified protocol was 96.6% while the recovery rate among those treated with the standard protocol was 87% [13]. In the latter group, children were required to recover based on two criteria (MUAC and WHZ), while recovery of children in the simplified protocol group was by MUAC only. Furthermore, in the standard protocol area, the number of discharge errors was higher, perhaps indicating greater challenges with the application of the standard protocol. Linked to this project, we conducted a costing study with the objective to determine the cost of acute malnutrition treatment in this area, as well as to investigate the influence of treatment protocol, programme size and severity of nutritional status on cost and cost-efficiency.

## 2. Materials and Methods

### 2.1. Methods

This costing study was conducted as part of an operational research project entitled “Increased coverage of management of acute malnutrition through the support of community health workers in emergency areas using a simplified or standard treatment protocol in Niger” conducted by Action Against Hunger, the Nutrition Direction of the Ministry of Health, CERMES and the EPINUT Research Group from the Complutense University of Madrid between November 2020 and July 2021. The project was approved by the National Health Research Ethics Committee of Niger with reference number 013/2020/CNERS.

This costing study was conducted from a societal perspective, which means that costs for treatment of acute malnutrition incurred by both the communities and project partners, namely village health posts, health centres, the district hospital, the regional health directorate, Action Against Hunger, UNICEF and the World Food Programme (WFP), were included.

### 2.2. Study Settings and Interventions

The study was carried out in the two adjacent municipalities of N’Guigmi and Kabléwa, in N’Guigmi district, Diffa region, Niger. Acute malnutrition prevalence among children under 5 years of age in N’Guigmi district was estimated to be 19.3% (15.9–23.2%) in 2020 [14]. In both study locations, treatment was provided at the community health centre and three village health posts. The health posts were staffed either by community health workers (CHW) or nurses. In each village, community health volunteers (CHV) known locally as “Relais Communautaires” also supported health post staff with detection of malnutrition, nutrition and health education as well as home visits for enrolled children who missed follow-up appointments. Coordination and supervision support were provided by the regional health directorate and the non-governmental organisations Action Against Hunger (at the health centre and health post level) and Médecins sans Frontières (at the district hospital). Treatment supplies including RUTF, ready-to-use supplementary food (RUSF) and medicines were provided by UNICEF and WFP.

Children admitted for treatment in N’Guigmi were treated according to a simplified treatment protocol and children admitted in Kabléwa were treated according to the standard national protocol. The two protocols differed in terms of admission and discharge criteria as well as type and dose of ready-to-use foods (RUF) (Table 1). In addition to different treatment protocols, the two areas also differed in population size, population density, number of admissions and treatment coverage [15,16,17] (Table 2).

### 2.3. Perspective and Time Horizon

The time horizon for this study was from November 2020 to the end of June 2021, and includes the training for health workers conducted at the start of November prior to enrolment of the first patients at the end of November.

### 2.4. Programme Data

Data on the number of admissions, number of RUTF or RUSF sachets provided and length of stay was obtained from the main study database. The methods and data on treatment outcomes of the main study have been published in detail elsewhere [13]. In brief, it was planned to include all children aged 6–59 months with moderate or severe acute malnutrition enrolled for treatment at one of the participating health centres or health posts during a 6-month period from November 2020 to April 2021. Study data collectors visited the health centres and posts regularly to retrieve data from health records on number of children enrolled, RUTF or RUSF sachets provided and length of stay as well as treatment outcomes of children admitted during the above-mentioned period. In total a full set of data were available for 1244 children, 899 in N’Guigmi and 355 in Kabléwa. Children in both areas had similar MUAC at admission, but children in Kabléwa had a lower mean weight-for-height z-score (WHZ). There was no significant difference in the proportion of MAM and SAM children. In both areas, the majority of children were treated at health centres (Table 3).

### 2.5. Cost Data Collection

Cost were collected using an activities and ingredients approach. An overview of activities and ingredients are shown in Table 4.

Costs were collected from Action Against Hunger accountancy records, key informant interviews and interviews with carers of children admitted for treatment. Data on the quantity of RUTF and RUSF consumed per child per treatment location was extracted from the study database. We included only country level costs, i.e., costs for coordination staff of organisations outside the country level and international transport costs for RUTF and RUSF were not included.

Action Against Hunger accountancy was obtained in January 2022, and all costs that were not directly linked to treatment of malnutrition or were outside the time horizon were excluded. Research costs were excluded where possible.

A total of 37 key informants were consulted in December 2021 either through individual or group interviews. Key informants included 13 CHVs, 6 health post staff (either CHWs or nurses), 6 health centre staff and 5 staff from the district referral hospital, as well as staff from Action Against Hunger, Médecins sans Frontières, and the regional Ministry of Health. UNICEF and WFP were later contacted via e-mail.

All health centres and health posts participating in the study were visited by the data collectors. The objective of the key informant interviews was to determine the number of individuals involved in the project and time spent on project activities as well as any other costs that could not be obtained from the accountancy data such as RUF costs (including transport) and costs linked to running a treatment programme at a health centre or health post. Interviews explored whether costs and time were linked to the research component of this study or would occur as part of a normal programme.

Lastly, structured interviews were conducted with carers of enrolled children, as part of a socio-economic survey, to determine the time needed for treatment, including transport and time spent at the health centre as well as cost of transport, accommodation and average salary of the main caregiver of the child. Due to security-related access issues, study staff were not able to visit the sites with the intended frequency, and, therefore, out of the 1244 children enrolled, only 871 caregivers (70%) responded to the survey. Family costs were calculated by adding expenses related to treatment (accommodation, food and transport) to time lost in wages. Time in lost wages in turn was calculated by multiplying the median hourly wage reported by the child’s primary caregiver by the time spent at the health centre and commuting.

### 2.6. Cost Data Management

Cost data from each data source—accountancy data, key informant interviews, caregiver interviews and the study database—were combined into an excel spreadsheet organised according to cost centres (Table 1). All costs are expressed in 2021 USD Dollars. Where costs were expressed in other currencies, these were converted to USD using average yearly exchange rates [18].

Costs were adjusted for inflation and capital cost depreciation. Adjusting for inflation was done using the Consumer Price Index [19]. Capital costs, defined as any item that can be used for more than one year and costs more than USD 100 [20], were adjusted to only reflect the cost of the time the item was used. For computers, printers and other technological supplies, a useful life of 5 years was assumed [21]. Salary costs, obtained either from Action Against Hunger accountancy or key informant interviews, were adjusted for time spent on the project. Health post and community health centre rental, upkeep and salary costs were adjusted according to the proportion of time spent on malnutrition activities according to key informants. Similarly, a proportion of Action Against Hunger office rental and coordination costs was allocated to this project based on the number of projects in the country. Total RUF costs were calculated by multiplying the average amount of sachets by cost per sachet.

While treatment costs were calculated separately for each study location, training, support, coordination and supervision costs needed to be allocated to the two areas. CHW training costs were allocated at 50% for each location, given that the same number of CHWs were trained in each arm. Support, supervision and coordination costs were allocated at 40% in Kabléwa and 60% in N’Guigmi as recommended by the study manager. Costs for mass screening activities were allocated to the two sites based on population size (35% of costs to Kabléwa and 65% to N’Guigmi).

### 2.7. Analyses

(i) Base case

In the base case analysis, we calculated total programme costs and average cost per fully treated child (*n* = 1244) and investigated the main drivers of treatment costs in the two locations. We also presented cost disaggregated for MAM and SAM children. Cost per child treated, which is a cost-efficiency measure, was calculated by dividing the total cost by the number of children treated.

(ii) Accounting for uncertainty

Areas of uncertainty during the cost data collection include the allocation of support, supervision and coordination costs; human resource costs as they relate to the time allocated to malnutrition treatment; health centre and health post costs, particularly where shadow rental prices were applied; and RUF costs.

In order to account for uncertainty in cost estimates, we used an approach based on triangular fuzzy numbers as described by Myatt et al. [22]. This approach is similar to a sampling-based approach but is based on a triangular distribution instead of binomial or probability distributions [22]. Triangular fuzzy numbers are expressed as a minimum, a maximum and a most likely value, where the most likely value is the one estimated by the data collection in the base case and the minimum and maximum were calculated as the base case ± the assumed margin of error. The margin of error applied to each cost centre along with a justification can be found in Supplementary Table S1. Based on the triangular fuzzy numbers, which can be thought of as the estimate and a 100% confidence interval (CI), we then calculated a 95% CI for costs using a fuzzy arithmetic calculator [23].

(iii) Sensitivity analyses

In addition to areas of uncertainty mentioned above s, uncertainty was also linked to the number of children treated. During the data collection phase, the teams managed to retrieve data from 1244 participants. Unfortunately, due to the COVID-19 pandemic, challenges in accessing the sites during the study period and the study finishing early due to security concerns, data collectors were not able to visit the sites with the intended frequency and mentioned that they were not able to collect data from all admitted children during the study period. Unfortunately, the exact amount of missing data is unknown. In order to understand what would happen if we had all the intended data, we conducted a sensitivity analysis with an additional 30% of children (*n* = 1617). In this sensitivity analysis, we recalculated the total programme costs, applying data on average RUF consumption to a greater number of children. Similarly, RUF transport costs were increased to reflect the larger number of children. Other costs such as coordination costs, facility costs and time spent on treatment by health workers were kept constant.

Furthermore, given the unequal number of children in the two areas, we carried out a modelled scenario sensitivity analysis with equal numbers of children in each location in order to better understand the impact of the different protocols and allocation of fixed costs on cost per child treated. In this analysis we increased the number of children treated and the number of health centre staff in the standard protocol area to the same numbers as the simplified protocol area. In the standard protocol area, the number of RUF sachets were increased by multiplying the average number of sachets consumed in this area by the greater number of children and transport costs were adjusted to reflect the increased amount of RUF boxes. Supervision, support, coordination and mass screening costs as identified in the accountancy were allocated at 50% each to the two locations in this analysis.

## 3. Results

### 3.1. Total Programme Costs

The total estimated cost of the intervention, treating both MAM and SAM, over the nine months intervention period in the base case scenario was estimated at ~USD 164,268.5 (95% CI: 149,560.9; 179,056.5) or USD 132 (95% CI: 120.2; 143.9) per child. Broken down into study locations, the cost was USD 66,483.1 (95% CI: USD 60,857.7; USD 72,136.2) in Kabléwa, which is equivalent to USD 187.3 (95% CI: USD 171.4; USD 203.2) per child treated, and ~USD 97,785.4 (95% CI: 88,703.12; 106,920.26) in the simplified protocol area (N’Guigmi), which corresponds to USD 110.2 (95% CI: USD 100.0; USD 120.3) per child treated (Table 5). The biggest cost contributors were coordination, support and supervision costs at 64.6% and 59.3% of total costs in Kabléwa and N’Guigmi, respectively, followed by RUF (RUSF and/or RUTF) costs, which accounted for 13% of total costs in Kabléwa and 14.4% in N’Guigmi (Table 5). While the proportion of RUF costs was higher in N’Guigmi, the cost of RUF (Table 5) and amount of RUF sachets consumed was lower in N’Guigmi, at a median of 42 (Inter-quartile range (IQR): 28–70) sachets per child compared to a median consumption of 60 (IQR: 45–74) sachets per child in Kabléwa. Household costs were USD 0.59 in Kabléwa and USD 0.81 in N’Guigmi. This was due to the low estimated hourly wage of caregivers (XOF 42.5) and close proximity of households to the study site, whereby 83% and 75% of the population in Kabléwa and N’Guigmi, respectively, lived within 45 min of the treatment site. The total time investments of participants for commute and time spent at health facilities for the entire duration of treatment was approximately 8 h in Kabléwa and 11 h in N’Guigmi.

### 3.2. Costs of MAM vs. SAM Treatment

When disaggregating into severity of malnutrition, the cost of treating a MAM child was USD 165.2 (95% CI: 151.7; 179.3), compared to USD 192.4 (95% CI: 175.9; 209.0) for treating SAM in Kabléwa using the standard protocol. In N’Guigmi, where the simplified protocol was used, treatment cost was USD 96.5 (95% CI: 87.3; 100.3), compared to USD 118 (95% CI: 107.2; 129.8) for MAM and SAM, respectively (Supplementary Table S2). While we did not estimate there to be a difference in training, coordination, support and supervision costs between MAM and SAM, the direct treatment costs (including staff time, location and RUTF) were approximately double for SAM compared to MAM (Supplementary Table S2). Specifically, in Kabléwa, the direct treatment cost of MAM and SAM was USD 28.2 (95% CI: 25.5; 31) and USD 54.9 (95% CI: 49.4; 60.4), respectively, whereas, in N’Guigmi, the cost of MAM treatment was USD 20.65 (95% CI: 18.5; 22.8) and SAM treatment costs were USD 42.5 (95% CI: 38; 47.1). This is partly due to higher dosages of RUF in SAM children. In Kabléwa, the estimated RUF costs per MAM child were USD 17.84, compared to USD 31 for a child with SAM, while, in N’Guigmi, the estimated RUTF costs per MAM child were USD 10.9, compared to USD 21.9 for a child with SAM (Supplementary Table S2).

### 3.3. Sensitivity Analysis

The sensitivity analysis shows that increasing the number of children reduces treatment costs, as the fixed costs—coordination and support costs—are spread over a greater number of children (Table 6). Similarly, in the modelled scenario sensitivity analysis, the cost of treatment in the standard protocol area and therefore the difference in costs between the two locations was reduced.

With regard to the difference between arms, in the base case analysis, the difference in cost per child treated is USD 77.3 (95%CI: USD 51.2; USD 103.4). Out of this, 69.6% of the difference (USD 53.8) is due to higher support and coordination costs per child, while 11% of the differences in costs (USD 8.5) is due to RUF costs. The difference in costs between the two groups is reduced from USD 77.3 to USD 61.5 in the sensitivity analysis with an additional 30% of children. This reduction in difference is mostly driven by lower coordination and support costs per child. In the modelled scenario sensitivity analysis, the difference between the two groups is further reduced (Table 6). In this scenario, the difference in costs is driven only by lower RUTF costs in children who were treated according to a simplified protocol (Table 6).

## 4. Discussion

The cost of acute malnutrition treatment in Diffa was USD 132 (95% CI: 120.2; 143.9) on average. Disaggregation of costs into the two locations found that costs were significantly higher in Kabléwa than N’Guigmi. Furthermore, MAM treatment was cheaper than SAM treatment: in Kabléwa, MAM treatment cost USD 165.5 (95% CI: 151.7; 179.3), compared to USD 192.5 (95% CI: 175.9; 209.0) for SAM treatment, while in N’Guigmi, MAM and SAM treatment cost USD 96.5 (95% CI: 87.3; 100.3) and 118.5 (95% CI: 107.2; 129.8), respectively. Our results are within the range of cost estimates found in previous studies. A study in Sierra Leone estimated the cost of MAM treatment with RUSF to be USD 105 per child [24], which falls within the range of MAM treatment costs found in Kabléwa and N’Guigmi. A study in Mali found that direct treatment costs—health centre staff, health centre infrastructure, medicine and RUSF—were USD 38.1 for treating a child with MAM with RUSF, while in our study direct treatment costs for MAM were only USD 28.24 in Kabléwa and USD 20.65 in N’Guigmi. Two previous cost analyses in Niger estimated the cost of treating a child with SAM to be USD 142 [25] or EUR 148.9 [12]. Overall, however, cost of SAM treatment in the literature varies widely, ranging from USD 56 to USD 805 [4,9,10,12,25,26,27,28,29,30,31].

The wide variation in treatment costs has previously been attributed to both differences in costing methods used and contextual determinants such as programme and population size [11]. The importance of context has also been demonstrated in our study, where higher treatment costs in Kabléwa were largely due to less admissions, 355 in Kabléwa compared to 899 in N’Guigmi, resulting from both lower population size and lower coverage. When programme size is low, fixed costs—training, coordination, supervision and infrastructure costs—are spread over a lower number of children, resulting in higher cost estimates. The impact of programme size and coverage is further highlighted by the results of our sensitivity analysis, which showed that increasing the number of children reduces costs per child treated, while having equal numbers of admissions in both locations results in similar costs in both areas. In line with our findings, a previous study in Niger found cost to be significantly influenced by programme size, with cost estimates ranging from EUR 49.7 for a site with 8321 admission per year to EUR 140.6 per year for a site with 996 admissions [12].

Within both study areas, the biggest drivers of costs were coordination, support and supervision costs, followed by RUTF costs, at 61% and 17% (if transport was included) of total costs. These findings are in line with other studies which used similar approaches to costing [4,9,10,24,31]. Naturally, in studies where coordination, support or infrastructure cost were not factored into the total costs, the proportion of RUTF costs was comparatively higher [12,32,33].

The RUF costs differed according to protocol used and severity of acute malnutrition: In N’Guigmi, where the simplified treatment protocol was used, RUF costs alone were estimated to be USD 15.9 per child, compared to USD 24.4 in Kabléwa, for treatment of both MAM and SAM children. This difference was more pronounced in SAM children only. The cost of treating one child with SAM was USD 31.4 in Kabléwa and USD 21.2 in the simplified protocol area. It is worth noting that the reduction in dose did not appear to have a negative impact on recovery rates [13]. The modelled scenario sensitivity analysis showed that, all other things being equal, the difference in RUF costs remains, thus providing robust evidence for cost savings in terms of RUF where the simplified protocol was used. Previous studies examining the effects of dose reduction and effectiveness have also yielded promising results: A study in the Democratic Republic of Congo found that applying the OPTIMA reduced dosage protocol led to significant reduction in RUTF costs, from USD 12,012 in the standard group (~USD 50 per child) to USD 6510 (~USD 26.9 per child) in the reduced dosage groups while showing similar recovery rates in both groups [5]. Similarly, a study in Burkina Faso found a reduction in RUTF costs of USD 15.4 per child treated when changing to a reduced dosage protocol [33]. A study conducted in Kenya and South Sudan, using very similar dosage protocols as reported here, found costs per child treated to be USD 435 with the combined protocol and USD 451 with the standard protocol. This difference was driven by a difference in RUTF costs [4]. The higher costs of RUF in Kabléwa compared to N’Guigmi found in our study can be explained by both the different dosage of RUTF in SAM children and longer lengths of stay in MAM children. In the simplified protocol area, children with SAM received two sachets of RUTF per day, while the Kabléwa children received a weight-based dosage of RUTF (Table 1). This resulted in a higher provision of RUTF to SAM children in Kabléwa compared to N’Guigmi at a median of 70 sachets compared to 95 sachets, respectively [13]. In addition to the dosage protocol, the higher amount of RUF, and thus RUF costs, is also due to longer lengths of stay, specifically in MAM children, where the length of stay was 14 days longer in the standard protocol area. This, in turn, is not surprising given the different exit criteria (Table 1), whereby children in Kabléwa had to recover according to both the MUAC and WHZ criteria while children in N’Guigmi had to recover only according to MUAC. The impact of the two criteria on length of stay is further increased by the fact that children in Kabléwa had a lower WHZ at admission.

This study had a number of limitations. First, challenges encountered during the data collection period—the COVID-19 pandemic, security, and access issues—led to data collectors being unable to regularly visit sites as planned and resulted in missing data. Furthermore, the socio-economic survey from which societal data were drawn was only answered by 70% of the caregivers. Second, we had to make a number of assumptions where accountancy data were not available and apply the shadow prices. Third, the RUF costs were based on the amount of RUF provided to children and did therefore not include losses or wastage.

Nevertheless, this study also has a number of important strengths. First, this data adds to the limited evidence base on the cost of CMAM programming, and, to our knowledge, it is one of very few studies that have costed the decentralised approach to treatment provided at the CHW or health post level in combination with a reduced dosage protocol. Secondly, this study demonstrates the important impact of context on costs and that costs can differ significantly even in neighbouring districts, as well as the influence of protocol and reduced RUF dosage on treatment costs. Furthermore, by aiming to include all children presenting at participating health centres and health posts, this cost-efficiency study aimed to give a picture of treatment costs under programme rather than research conditions, though we have to acknowledge that it can be challenging to disentangle research costs from programming costs in an operational research project. We conducted a sensitivity analysis to understand the impact that any missing data may have had on treatment costs as well as a modelled scenario sensitivity analysis to understand the impact of the protocol on costs had the location and caseload been the same. Finally, our study has identified the main cost drivers, which include coordination and RUTF costs, as well as coverage and severity of malnutrition.

Future research should assess cost-effectiveness in addition to cost-efficiency and could explore other approaches to reduce treatment costs without sacrificing effectiveness. Several studies have already investigated reducing RUTF costs by either reducing dosage or altering formulations of RUTFs, for example, by replacing expensive ingredients such as milk with legumes. In addition, our findings suggest that exploring ways of reducing coordination, support and supervision costs while maintaining programme effectiveness and adequate levels of supervision may be a good way to reduce treatment costs. As our results have shown that direct treatment costs for treatment of SAM were roughly double those of MAM, future research may want to model whether increasing access to MAM treatment and treating malnutrition earlier, for example, through combined treatment approaches, can lead to cost savings in the long term despite the higher caseloads.

## 5. Conclusions

In conclusion, our study has shown that the cost of acute malnutrition treatment in Niger is ~USD 132 and highlighted the impact of treatment protocol and context on cost. Results suggest that the cost of acute malnutrition treatment can be reduced by treating malnutrition earlier, increasing coverage and by using a simplified protocol with a modified dosage of RUTF and only one admission and discharge criterion. However, more evidence is needed with regards to cost-effectiveness of the simplified protocol as well as approaches to reduce treatment costs, particularly targeting coordination, supervision and support costs, which were the biggest contributor to overall costs.

## Figures and Tables

**Table 1:** Overview of treatment protocols in the two study areas.

	Kabléwa (National Protocol)	N’Guigmi (Simplified Protocol)
Admission criteria	WHZ < −2 or MUAC < 125 mm or oedema +/++	MUAC < 125 mm or oedema +/++
Treatment product and dose	Children with SAM: 170 kcal/kg/day of RUTF Children with MAM: 1 sachet of RUSF/day	Children with SAM: 2 sachets of RUTF/day * Children with MAM: 1 sachet of RUTF/day
Discharge criteria (2 consecutive visits)	WHZ ≥ −2 and MUAC ≥ 125 mm without oedema	MUAC ≥ 125 mm without oedema

* Children under 5 kg received just 1 sachet/day (to not exceed the quantity recommended by the national protocol).

**Table 2:** Characteristics of the two study areas.

	Kabléwa (National Protocol)	N’Guigmi (Simplified Protocol)
Total population ^1^	30,475	54,950
Population density ^2^	4 people/km^2^	15 people/km^2^
Treatment protocol	National protocol	Simplified protocol
MAM Coverage ^3^	November 2020: 25.30% (25.1–25.5%) December 2021: 13.60% (13.4–13.9%)	November 2020: 28.80% (28.5–29%) December 2021: 84.9 (84.8–85.1%)
SAM Coverage ^3^	November 2020: 29.60% (28.9–30.4%) December 2021: 65.20% (64.2–66.2%)	November 2020: 35.90% (35.3–36.5%) December 2021: 88.00% (87.6–88.4%)

^1^ Institut National de la Statistique, Ministère du Plan, République du Niger. Annuaire statistique régional de Diffa 20,132,017 [Internet]. 2018; ^2^ Thomas Brinkhoff. City population: Niger. City population. [Internet] Available from: https://www.citypopulation.de (accessed on 10 February 2023); ^3^ Ousmane Ali. Enquête de couverture sur la prise en charge de la malnutrition aigue à N’Guigmi et Kablewa. 2021.

**Table 3:** Programme data in the two study locations.

	Kabléwa (*n* = 355)	N’Guigmi (*n* = 889)
Sex, % girls (*n*)	52.1 (185)	53.4 (475)
Age months (Median, IQR)	12 (9–15)	12 (9–17)
***Nutritional status at admission***		
MUAC in mm, mean (SD)	115.3 (5.7)	115.5 (4.9)
WHZ, mean (SD)	−2.66 (1.01) ^2^	−2.38 (1.26) ^2^
MAM ^1^, %(*n*)	50.9 (181)	54.3 (483)
SAM ^1^, % (*n*)	49 (174)	45.7 (406)
***Treatment site***		
Health post, % (*n*)	42.3 (150)	36.3 (323)
Health centre, % (*n*)	57.7 (205)	63.7 (566)

^1^ As per criteria in the district, i.e., case definition based on MUAC only in N’Guigmi and based on MUAC and weight-for-height in Kabléwa; ^2^ Difference between the two locations significant at the 0.05 level.

**Table 4:** Activity and ingredient costs included in the economic analysis.

Activity	Ingredients	Source
1. Training costs	Per diems for participants and trainers Transport costs (participants and trainers) Training materials Room rentals	- Action Against Hunger accountancy
2. Coordination, supervision and support	Staff salaries Per diems Transport costs Office costs (rental, upkeep, materials, capital costs)	- Action Against Hunger accountancy - Key informant interviews
3. Case finding	Community volunteers time Mass screening campaign	- Action Against Hunger accountancy - Key informant interviews
4. Direct treatment costs		
4.1 Health Centre	Health centre staff time Health centre rental and upkeep Nutrition treatment materials and equipment RUTF/RUSF, medicines and associated transport costs	- Action Against Hunger accountancy - Key informant interviews - Programme data
4.2 Health post	Health post staff time Health post rental and upkeep Nutrition treatment materials and equipment RUTF/RUSF, medicines and associated transport costs	- Action Against Hunger accountancy - Key informant interviews - Programme data
4.3 Referral to hospital	Referral transport Treatment costs including hospital rental, upkeep, staff, equipment, materials, RUTF and medicines	- Key informant interviews - Programme data
5. Family/Patient costs	Transport costs Food/accommodation costs Time in lost wages	Socio-economic survey among caregivers

**Table 5:** Intervention costs (USD) in the base case in Kabléwa (standard protocol) and N’Guigmi (simplified protocol) district, Niger.

Activity	Ingredients	Kabléwa (Standard Protocol Area)			N’Guigmi (Simplified Protocol Area)		
		Total Cost (USD)	% of Total Cost	Cost Per Child (USD)	Total Cost (USD)	% of Total Cost	Cost Per Child (USD)
1. Training	Staff, room rental, per diem, transport and training materials	2951.8	4.4%	8.31	2951.8	3.0%	3.32
2. Coordination, support, supervision and office	Staff, office, perdiem, transport	42,267.5	64.6%	119.06	57,996.2	59.3%	65.24
3. Case finding	Mass screening and community health volunteer time	3294.4	5.0%	9.28	5782.1	5.9%	6.5
4. Direct treatment	Health post staff time	1180.1	1.8%	7.87 *	944.1	0.9%	2.92 *
	Health centre staff time	1939.4	2.9%	9.46 *	1379.7	1.4%	2.44 *
	Health post rental and upkeep	569.8	0.9%	3.8 *	1893.3	1.9%	5.86 *
	Health centre rental and upkeep	1510.5	2.3%	7.37 *	5900.4	6.0%	10.42 *
	Treatment materials and equipment	1426.4	2.1%	4.02	1426.4	1.5%	1.6
	RUTF/RUSF costs	8668.44	13.3%	24.4	14,155.7	14.5%	15.9
	Medicines	180.3	0.3%	0.51	346.3	0.4%	0.39
	RUTF/RUSF transport	2311.27	3.4%	6.51	4289.5	4.4%	4.8
5. Societal costs		209.45	0.3%	0.59	720.09	0.74%	0.81
Total costs		66,483.16	100%	184.1	97,785.40	100%	110.2

* Costs are per child treated at health post and health centres respectively. In Kabléwa, out of the 355 children, 150 were treated at health posts and 205 at the health centre. In N’Guigmi, out of the total of 889 children, 323 children were treated at health posts and 566 at the health centre.

**Table 6:** Cost per child treated in the sensitivity analyses with an increased number of children and a modelled scenario.

Activity	Sensitivity Analysis with Increased Number of Children Treated			Modelled Scenario Sensitivity Analysis		
	Kabléwa (*n* = 462)	N’Guigmi (*n* = 1155)	Δ	Kabléwa (*n* = 889)	N’Guigmi (*n* = 889)	Δ
Training, USD	6.39	2.55	3.84	3.32	3.32	0
Coordination, support and supervision, USD	91.49	50.17	41.32	56.39	56.39	0
Case finding, USD	7.13	5.00	2.13	5.16	5.16	0
Health post and health centre staff, USD	6.75	2.01	4.74	3.51	2.61	0.9
Health post and health rental and upkeep, USD	4.50	6.74	−2.24	8.77	8.77	0
Treatment materials, USD	3.09	1.23	1.86	1.60	1.60	0
RUTF and RUSF costs, USD	24.39	15.92	8.47	24.33	15.92	8.41
Medicines, USD	0.51	0.39	0.12	0.49	0.39	0.1
Logistics and RUTF/RUSF transport costs, USD	5.37	4.10	1.27	5.80	3.69	2.11
Societal costs, USD	1.12	1.14	−0.02	1.12	1.00	0.12
Cost per child, USD (95%CI)	150.74 (138.3; 165.6)	89.26 (81.2; 98.7)	61.5 (41.6; 82.5)	110.49 (100.4; 120.6)	98.87 (89.8; 107.9)	11.6 (−3.2; 26.5)

## Data Availability

The data presented in this study are available upon request from the corresponding author.

## Supplementary Materials

The following supporting information can be downloaded at: https://www.mdpi.com/article/10.3390/nu15173833/s1, Table S1: Cost per child treated (detailed); Table S2: Treatment costs disaggregated by MAM and SAM.
