# Supplementary material for: Cost of Acute Malnutrition Treatment Using a Simplified or Standard Protocol in Diffa, Niger
Source: Nutrients. 2023 Sep 1;15(17):3833. doi: 10.3390/nu15173833 (PMC10490076; doi:10.3390/nu15173833)
Supplement: Supplementary file 1 [file nutrients-15-03833-s001.zip › nutrients-2526923-supplementary.pdf]

Supplementary Table S1. Cost per child treated (detailed).

Activity	Ingredients	Margin of Error and Justification	Standard Protocol Area			Simplified Protocol Area		
			Lower Limit	Base Case	Upper Limit	Lower Limit	Base Case	Upper Limit
**1. Training**	Staff costs, room rental, per diem, transport and training materials	Treated as certain because these costs have been taken straight from the accountancy.	2951.80	2951.80	2951.80	2951.80	2951.80	2951.80
**2. Coordination, support, supervision and institutional support**	2.1. Personnel - AAH	Treated as uncertain, given that % of staff time spent on project activities is an estimate as is the allocation between arms. We assumed a 20% margin of error.	11124.30	13905.37	16686.45	16686.45	20858.06	25029.67
	2.2. Consultancies (Coverage)	Treated as certain because price is fixed and as found in accountancy.	10809.97	10809.97	10809.97	10809.97	10809.97	10809.97
	2.2. Office costs - AAH	Treated as uncertain. Uncertainty linked to the cost allocated to the project and to the uncertainty of allocation between arms. We assumed a 10% margin of error.	3612.21	4013.57	4414.92	5418.31	6020.35	6622.38
	2.3. Per diem and food costs -AAH	Costs came from the accountancy data. But the allocation between study arms which was made at 40% to the control and 60% to the intervention area was uncertain. We assumed a 10% margin of error.	2515.26	2794.73	3074.20	3772.88	4192.09	4611.30
	2.4. Transport costs - AAH	Costs were taken from the accountancy data. But the allocation between study arms which was made at 40% to the control and 60% to the intervention area was uncertain. We assumed a 10% margin of error.	8748.97	9721.08	10693.19	13123.46	14581.62	16039.78
	2.5. DRSP staff costs	Uncertainty stems from the % of time spent on nutrition activities as well as the allocation between study arms. We assumed a margin of error of 20%	475.80	594.76	713.71	713.71	892.13	1070.56

	2.6. Other DRSP supervision costs	Uncertainty stems from the % of time spent on nutrition activities as well as the allocation between study arms. We assume a margin of error of 20%	342.38	427.97	513.57	513.57	641.96	770.35
**3. Case finding**	3.1. Mass screening	Total cost was found in the accountancy data and is therefore fixed, however the allocation to study area (which was based on population size) is uncertain. We assume a margin of error of 10%	2504.70	2783.00	3061.30	4651.59	5168.44	5685.28
	3.2. Community volunteers (time)	Uncertainty stems from the % of time spent on nutrition activities in these two areas as well as the allocation between study arms. We assume a margin of error of 20%.	409.09	511.36	613.64	490.91	613.64	736.36
**4. Health post site**	4.1. Health post staff (time) on malnutrition treatment	Uncertainty stems from the amount of time spent on nutrition activities. We assumed a margin of error of 20%.	944.06	1180.07	1416.08	755.24	944.06	1132.87
	4.2. CHW sites costs (rental and upkeep)	Uncertainty stems from the fact that rental costs as well as upkeep costs and times spent on malnutrition activities were estimated. We assume an error margin of 20%	455.83	569.79	683.75	1514.61	1893.27	2271.92
	4.3. CHW materials	Fixed (from accountancy data)	570.88	570.88	570.88	570.88	570.88	570.88
	4.4.1. Cost of RUTF/RUSF a (MAM)	Numbers based on what was recorded for children with a full set of data. Assume margin of error of 10%	798.34	887.04	975.74	1097.71	1219.68	1341.65
	4.4.2. Cost of RUTF SAM	As above	2043.36	2270.40	2497.44	3970.89	4412.10	4853.31
	4.4.3. Cost of drugs MAM	Numbers based on what was recorded for children with a full set of data. Assume margin of error of 10%	1.01	1.12	1.23	17.94	19.93	21.93
	4.4.4. Cost of drugs SAM	As above	67.50	75.00	82.50	115.72	128.58	141.44
**5. Health centre**	5.1. Health centre staff (MAM and SAM)	Uncertainty stems from the amount of time spent on nutrition activities. We assumed a margin of error of 20%.	1551.48	1939.35	2327.22	1103.73	1379.67	1655.60

	5.3. Health centre rental and upkeep	Uncertainty stems from the fact that rental costs as well as upkeep costs and times spent on malnutrition activities were estimated. We assumed an error margin of 20%	1208.39	1510.49	1812.59	4720.28	5900.35	7080.42
	5.4. Health centre material and equipment	Fixed (from accountancy data)	855.53	855.53	855.53	855.53	855.53	855.53
	5.5.1. RUTF/RUSF at health centre (MAM)	Numbers based on what was recorded for children with a full set of data. We assume margin of error of 10%.	1140.48	1267.20	1393.92	3648.65	4054.05	4459.46
	5.5.2. RUTF/RUSF at health centre (SAM)	As above	2809.62	3121.80	3433.98	4022.87	4469.85	4916.84
	5.5.3. Medicines at Health centre (MAM)	Numbers based on what was recorded for children with a full set of data. We assumed margin of error of 10%.	27.39	27.39	35.60	53.01	53.01	68.91
	5.5.4. Medicines at health centre (SAM)	Numbers based on what was recorded for children with a full set of data. We assumed margin of error of 10%.	76.75	76.75	99.77	144.74	144.74	188.16
**6. Logistics costs**	6.1. Supply transport from Niamey to district (UNICEF)	We assumed a margin of error of 20% because the transport could have been with a different lorry (i.e. a bigger one that's cheaper)	518.07	647.58	777.10	1199.89	1499.86	1799.83
	6.2. ACF transport costs for buffer stock	Fixed costs, but allocation uncertain. We assumed a 10% margin of error.	150.93	167.70	184.47	352.17	391.30	430.43
	6.2. Transport costs District -> Health facility-> Health post	Fixed costs, but allocation uncertain. We assumed 10% margin of error.	1322.96	1469.96	1616.95	2158.52	2398.35	2638.19
**7. Referrals**			0.00	0.00	0.00	0.00	0.00	0.00
**8. Societal costs**			188.51	209.45	230.40	648.08	720.09	792.10
Total costs, with uncertainty range			58225.55	65361.11	72527.90	86083.11	97785.35	109546.91
Total costs, with 95%CI *			59822.85	65361.11	70927.10	88703.12	97785.35	106920.26
Cost per child, with uncertainty range			**164.02**	**184.12**	**204.30**	**96.83**	**109.99**	**123.22**
Cost per child, with 95%CI *			**168.51**	**184.12**	**199.79**	**99.77**	**109.99**	**120.27**

* Calculated using(http://www.brixtonhealth.com/fuzzy.html)

Supplementary Table S2. Treatment costs disaggregated by MAM and SAM.

**Activity**	**Ingredients**	**Kabléwa**		**N'Guigmi**	
		**MAM**	**SAM**	**MAM**	**SAM**
Training, coordination, support, supervision and case finding		136.65	136.65	75.06	75.06
Direct treatment costs	Cost of health post and health centre staff time	4.89	9.62	2.10	4.60
	Health post and health centre site costs (rental and upkeep)	3.67	8.14	5.84	12.25
	Treatment materials and equipment	1.10	2.10	0.50	0.80
	RUTF costs	0.00	31.41	10.92	21.88
	RUSF costs	17.84	0.00	0.00	0.00
	Medicine costs	0.09	0.92	0.13	0.67
	RUTF/RUSF transport costs	0.65	2.73	1.16	2.31
Direct treatment cost total		28.24	54.92	20.65	42.5
Societal costs		0.62	0.85	0.81	0.95
Total Cost per child treated		165.5	192.4	96.5	118.5
